# Efficacy and safety of adding immune checkpoint inhibitors to first-line standard therapy for recurrent or advanced cervical cancer: a meta-analysis of phase 3 clinical trials

**DOI:** 10.3389/fimmu.2024.1507977

**Published:** 2024-12-06

**Authors:** Xinmiao Zhang, Jinhai Shen, Mengfan Huang, Rongxia Li

**Affiliations:** ^1^ College of Integrated Traditional Chinese and Western Medicine, Hebei University of Chinese Medicine, Shijiazhuang, Hebei, China; ^2^ Department of Gynecology, The Second Hospital of Hebei Medical University, Shijiazhuang, Hebei, China; ^3^ State Key Laboratory of Natural Medicines, China Pharmaceutical University, Nanjing, Jiangsu, China; ^4^ Center for New Drug Safety Evaluation and Research, China Pharmaceutical University, Nanjing, Jiangsu, China

**Keywords:** immune checkpoint inhibitors, cervical cancer, efficacy, safety, meta-analysis

## Abstract

**Background:**

Immune checkpoint inhibitors (ICIs) combined with standard therapy (ST) have emerged as a novel treatment strategy for recurrent or advanced cervical cancer (r/a CC). However, the available data from phase 3 clinical trials have yielded mixed results. This study aims to evaluate the therapeutic efficacy and safety of adding ICIs to ST in the treatment of r/a CC.

**Methods:**

Data from four phase 3 clinical trials (KEYNOTE-826, CALLA, BEATcc, and ENGOT-cx11/GOG-3047/KEYNOTE-A18), involving 2,857 patients, were analyzed. Meta-analyses were conducted to combine hazard ratios (HRs) for overall survival (OS) and progression-free survival (PFS), odds ratios (ORs) for the objective response rate (ORR), and relative risks (RRs) for adverse events (AEs).

**Results:**

The addition of ICIs to ST significantly improved PFS (HR, 0.67; 95% CI, 0.60-0.75), OS (HR, 0.66; 95% CI, 0.58-0.75), and ORR (OR, 1.48; 95% CI, 1.13-1.94) compared to ST alone. However, there was a modest increase in grade 3-5 AEs (RR, 1.08; 95% CI, 1.03-1.13) with the combined therapy.

**Conclusion:**

This meta-analysis indicates that the combination of ICIs with ST in the treatment of r/a CC not only demonstrates superior efficacy over ST alone but also maintains a comparable toxicity profile, offering strong evidence for an effective and relatively safe treatment approach for managing this disease.

**Systematic Review Registration:**

https://www.crd.york.ac.uk/prospero/, identifier CRD42024593895.

## Introduction

Cervical cancer (CC) remains a significant global health issue, being the fourth most common cancer in women worldwide (1). For patients with recurrent or advanced cervical cancer (r/a CC), current standard therapies (ST), such as chemotherapy (CT), targeted therapy, and concurrent chemoradiotherapy (CCRT), offer limited benefits, underscoring the need for innovative treatments (2–4). Immunotherapy, especially immune checkpoint inhibitors (ICIs), has shown promise in various cancers and is increasingly being explored for CC treatment (5–8).

Recently, four phase 3 clinical trials have assessed the incorporation of ICIs into first-line ST for r/a CC. The KEYNOTE-826 trial evaluated the efficacy of pembrolizumab in combination with CT as a first-line treatment for r/a CC (9, 10). The results demonstrated a significant improvement in both progression-free survival (PFS) and overall survival (OS) compared to CT alone. This finding underscores the potential role of immunotherapy in the treatment of CC and supports its consideration in clinical practice. The CALLA trial investigated the addition of durvalumab to CCRT for the treatment of locally advanced cervical cancer (la CC) (11). Unfortunately, the study did not meet its primary endpoints of improving PFS or OS compared to CCRT alone. These results indicate that further research is necessary to determine the role of immunotherapy in the context of la CC treatment. The BEATcc trial assessed the efficacy of atezolizumab combined with platinum-based CT and bevacizumab as a first-line treatment for metastatic (stage IVB), persistent, or recurrent CC (12). The results showed a significant improvement in PFS and OS compared to CT and bevacizumab alone. This outcome highlights the potential of immunotherapy, in conjunction with traditional CT and targeted therapy, as a valuable treatment option for r/a CC, supporting its integration into clinical practice. The ENGOT-cx11/GOG-3047/KEYNOTE-A18 trial evaluated the therapeutic potential of combining pembrolizumab with CCRT as a first-line treatment for newly diagnosed, high-risk la CC (13, 14). The findings revealed a notable enhancement in both PFS and OS for patients receiving combination therapy compared to those treated with CCRT alone. These results reinforce the idea that immunotherapy plays a crucial role in the management of la CC and suggest its integration into standard clinical care. While KEYNOTE-826, BEATcc, and ENGOT-cx11/GOG-3047/KEYNOTE-A18 yielded positive results regarding their design, CALLA failed to meet its primary endpoint in the intention-to-treat (ITT) population. Additionally, the individual studies lacked sufficient power to analyze various clinically relevant subgroups.

Given the need in the treatment landscape of r/a CC, we conducted a meta-analysis of phase 3 clinical trials comparing the combination of ICIs with ST *versus* ST alone in patients with r/a CC. This study aims to provide insights into the clinical benefits and risks associated with the addition of ICIs to ST, empowering clinicians with robust data to inform their treatment decisions and patient management strategies.

## Methods

### Data sources

For this meta-analysis, data were sourced from four published phase 3 clinical trials: KEYNOTE-826, CALLA, BEATcc, and ENGOT-cx11/GOG-3047/KEYNOTE-A18. We extracted necessary data directly from the original publications of these trials and cross-referenced it with information available in clinical trial registries such as *ClinicalTrials.gov* to ensure consistency in trial design and reporting of outcomes. To guarantee the accuracy and completeness of the data, we also reviewed associated conference abstracts and supplementary materials. The data included primary and secondary endpoint results, along with key metrics for assessing treatment efficacy and safety.

### Data extraction and assessment of risk of bias

Data pertinent to the study objectives were extracted by a primary investigator and subsequently verified for accuracy by an independent secondary reviewer. The information extracted included, where available, the title of the clinical trial, the date of publication, sample size, study design, therapeutic regimens for both the experimental and control groups, characteristics of the participants, hazard ratios (HRs) along with their corresponding 95% confidence intervals (CIs) for OS and PFS, odds ratios (ORs) with associated 95% CIs for objective response rate (ORR), and the incidence of any adverse events (AEs), as well as the number of patients experiencing grade 3-5 AEs. The assessment of risk of bias was conducted using the Cochrane Collaboration’s Risk of Bias assessment tool (15).

### Statistical analysis

For the efficacy analysis, HRs with 95% CIs for OS and PFS, as well as ORs with 95% CIs for ORR, were computed for each study to derive an overall estimate. In the context of safety analysis, relative risks (RRs) with 95% CIs for AEs were calculated for each study to obtain a comprehensive estimation. The *I²* statistic and the Cochrane *Q* test were utilized to evaluate between-study heterogeneity. An *I²* value exceeding 50% and a *p*-value below 0.1 from the *Q* test signified considerable heterogeneity, necessitating the use of a random-effects model. In contrast, a fixed-effect model was applied when these criteria were not satisfied. A funnel plot was created, and Egger’s test was conducted to evaluate publication bias. All statistical analyses were conducted using R (v4.2.2), with statistical significance set at a two-tailed *p*-value < 0.05.

## Results

### Characteristics of the four phase 3 trials

Among the four included studies, one employed an open-label design, while the remaining three utilized a double-blind design. A cumulative total of 2,857 patients diagnosed with r/a CC were analyzed. Of these, 1,428 patients were treated with ICIs in combination with ST, and 1,429 patients received ST alone. The experimental treatment regimens comprised pembrolizumab, durvalumab, and atezolizumab, each in conjunction with ST. The control arm regimens consisted of placebo plus ST, which included platinum-based CT ± bevacizumab, CCRT, and bevacizumab plus CT. The main characteristics of the four trials are summarized in **Table 1**.

**Table 1:** Main characteristics of the four phase 3 trials.

Study	Year	ITT population	Design	Age median, range, (IQR), years	Regimens		Population characteristics	Cancer stage at diagnosis
					Experimental arm	Control arm		
KEYNOTE-826 (9, 10)	2021 2023	617 ICIs + ST: 308 ST: 309	Phase III double-blind RCT randomization 1: 1	ICIs + ST: 51, 25-82 ST: 50, 22-79	Pembrolizumab plus platinum-based CT ± bevacizumab	Placebo plus platinum-based CT ± bevacizumab	Persistent, recurrent, or metastatic	I-IVB
CALLA (11)	2023	770 ICIs + ST: 385 ST: 385	Phase III double-blind RCT randomization 1: 1	ICIs + ST: 50 (41-57) ST: 48 (40-57)	Durvalumab plus CCRT	Placebo plus CCRT	Locally advanced	IB2-IVA
BEATcc (12)	2024	410 ICIs + ST: 206 ST: 204	Phase III open-label RCT randomization 1: 1	ICIs + ST: 51, 24-90, (43-60) ST: 52.5, 21-79, (43.5-61)	Atezolizumab plus bevacizumab and CT	Bevacizumab and CT	Metastatic, persistent, or recurrent	I-IVB
KEYNOTE-A18 (13, 14)	2024	1060 ICIs + ST: 529 ST: 531	Phase III double-blind RCT randomization 1: 1	ICIs + ST: 49 (40-57) ST: 50 (41-59)	Pembrolizumab plus CCRT	Placebo plus CCRT	Newly diagnosed, high-risk, locally advanced	IB2-IVA

ITT, intention-to-treat; IQR, inter-quartile range; ICIs, immune checkpoint inhibitors; ST, standard therapy; RCT, randomized clinical trial; CT, chemotherapy; CCRT, concurrent chemoradiotherapy.

### Efficacy analysis

#### PFS in ITT population

**Figure 1:**
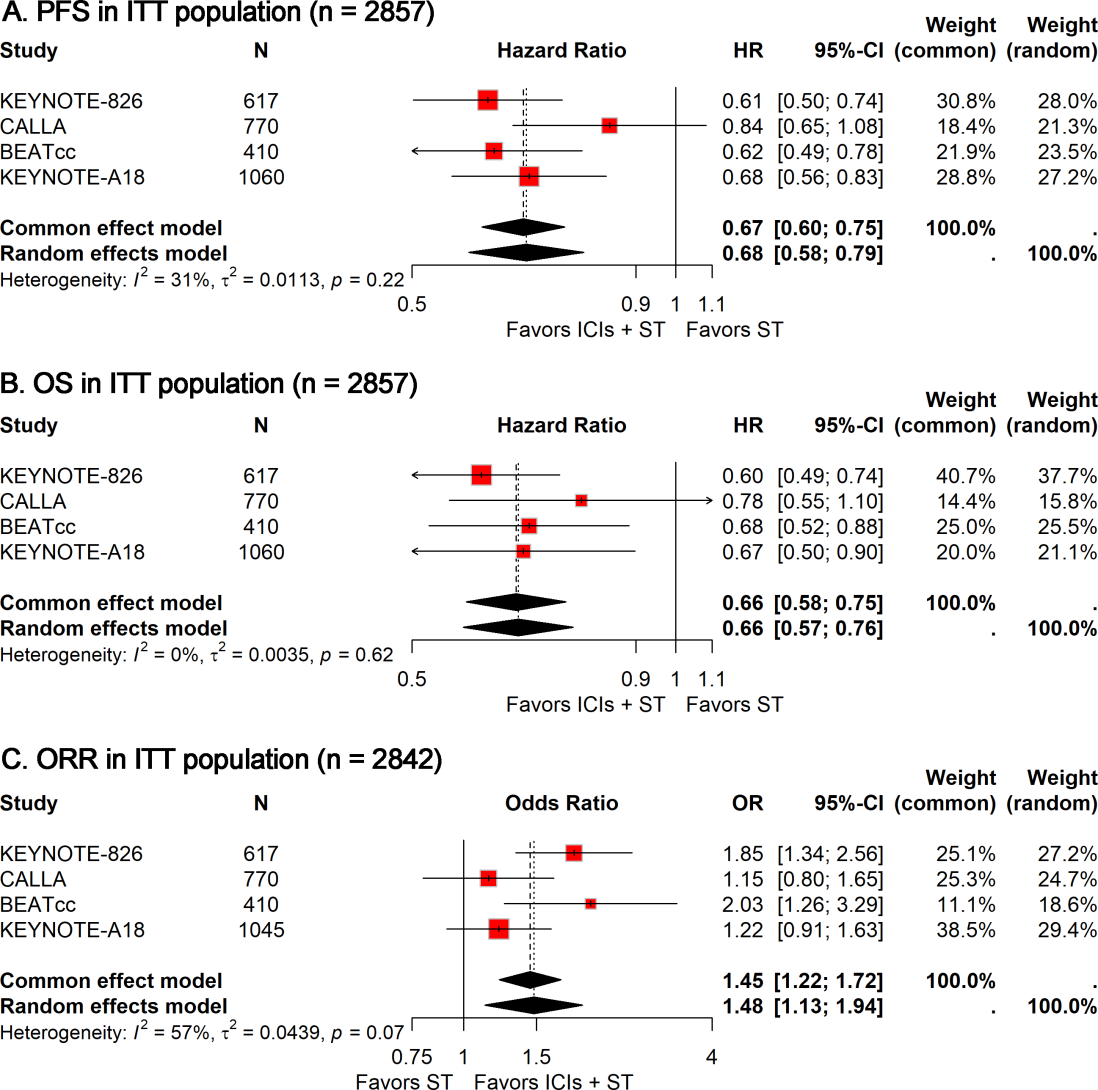
Forest plots comparing ICIs plus ST to ST alone in the ITT population for PFS **(A)**, OS **(B)**, and ORR **(C)**. PFS, progression-free survival; OS, overall survival; ORR, objective response rate; ICIs, immune checkpoint inhibitors; ST, standard therapy; ITT, intention-to-treat.

In the absence of significant between-study heterogeneity (*I²* = 31%), a fixed-effect model was utilized to derive the pooled estimate of PFS. The combined analysis showed that adding ICIs to ST significantly improved PFS compared to ST alone (HR, 0.67; 95% CI, 0.60-0.75; **Figure 1A**).

#### OS in ITT population

Similarly, no heterogeneity (*I²* = 0) was observed across these studies. The meta-analysis suggested that combining ICIs with ST led to a significant extension of OS compared to ST alone (HR, 0.66; 95% CI, 0.58-0.75; **Figure 1B**).

#### ORR in ITT population

Given the significant heterogeneity observed among studies (*I²* = 57%), a random-effects model was used to compute the combined OR with 95% CI. The meta-analysis suggested that the addition of ICIs to ST significantly enhanced the ORR compared to ST alone (OR, 1.48; 95% CI, 1.13-1.94; **Figure 1C**).

### Safety analysis

#### Grade 3-5 AEs

**Figure 2:**
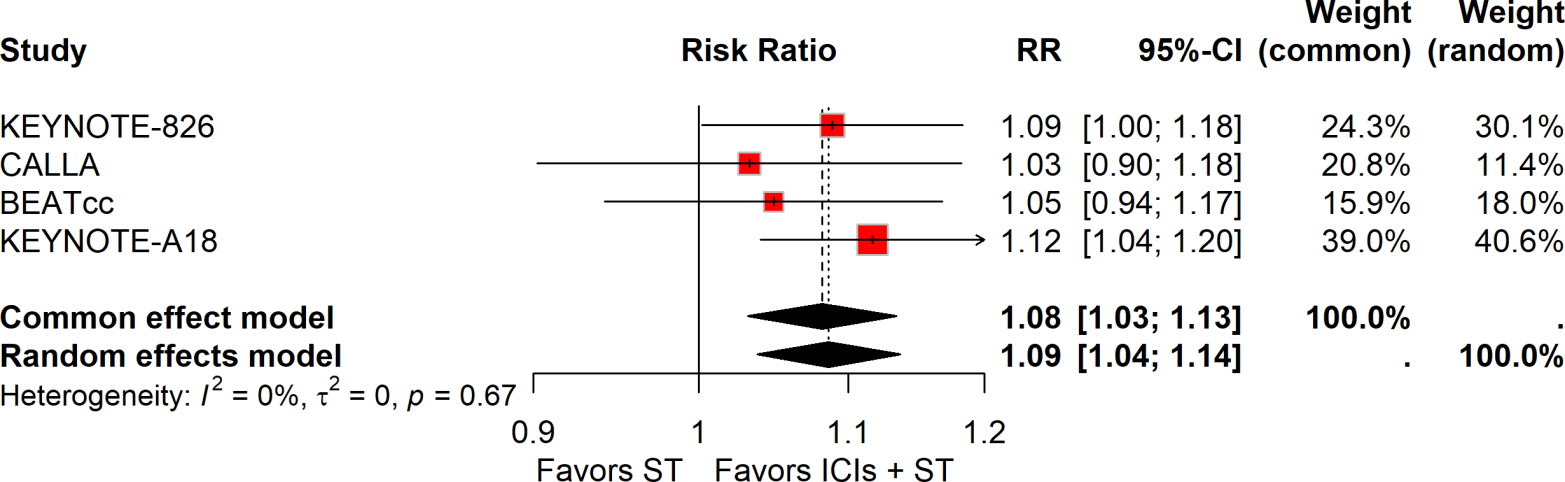
Forest plot of grade 3-5 AEs comparing ICIs plus ST to ST alone in the ITT population. AEs, adverse events; ICIs, immune checkpoint inhibitors; ST, standard therapy; ITT, intention-to-treat.

Among the 1,425 patients treated with ICIs plus ST, 1,029 (72.2%) experienced grade 3-5 AEs, compared to 949 out of 1,422 patients (66.7%) in the ST alone group. The meta-analysis showed that adding ICIs to ST was linked to a small rise in the risk of grade 3-5 AEs (RR, 1.08; 95% CI, 1.03-1.13; **Figure 2**).

### Subgroup analysis

To achieve a more profound understanding of the efficacy of ICIs combined with ST in patients with r/a CC, we conducted several stratified analyses based on patient characteristics and treatment regimens.

**Figure 3:**
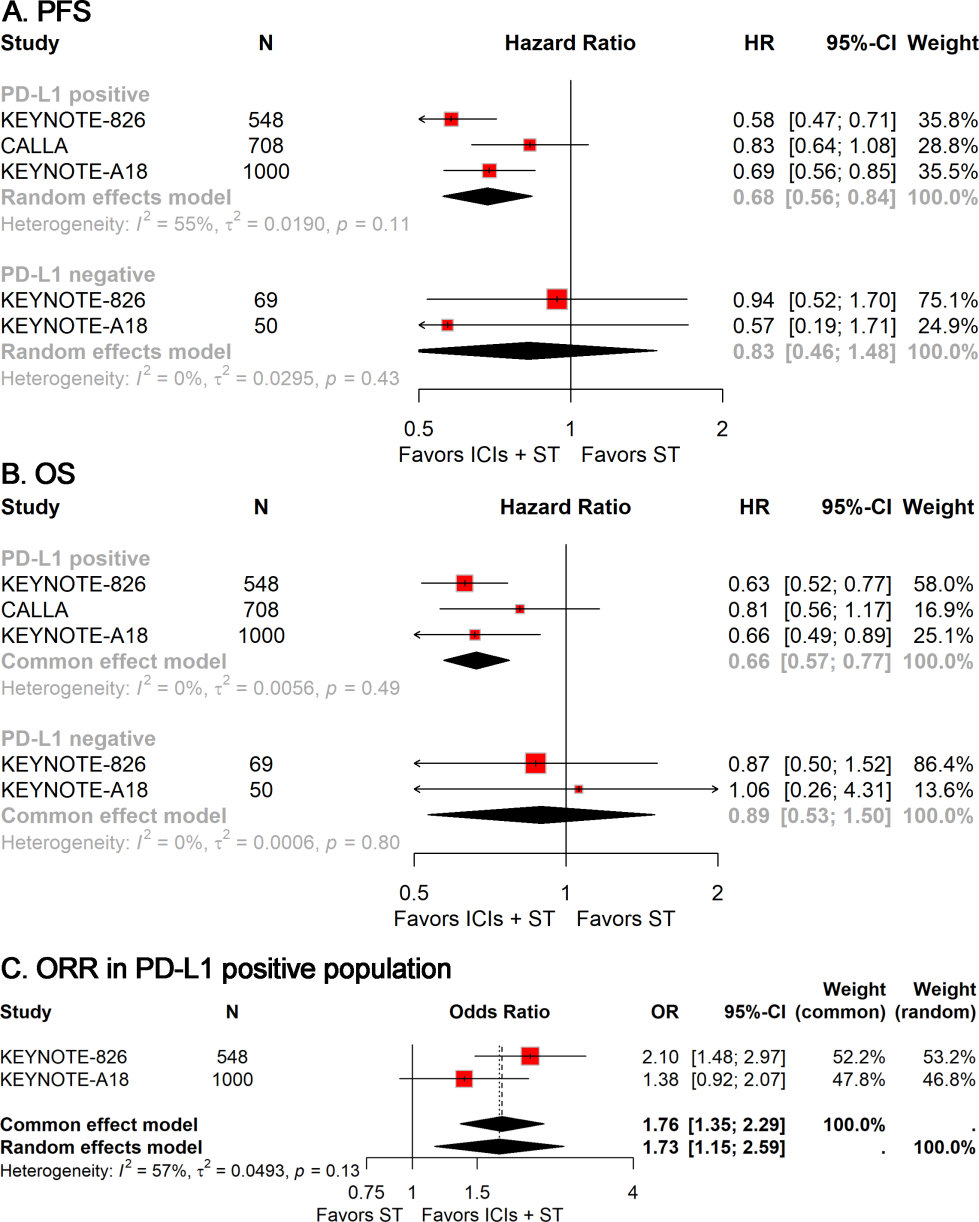
Forest plots of subgroup analysis stratified by PD-L1 status, showing results for PFS **(A)**, OS **(B)**, and ORR **(C)**. PFS, progression-free survival; OS, overall survival; ORR, objective response rate.

In light of the observed heterogeneity within the r/a CC cohort and significant variations based on PD-L1 status, we performed a targeted subgroup analysis to ascertain whether PD-L1 status could serve as a biomarker for the efficacy of ICIs plus ST. The analyses revealed that the combination of ICIs with ST significantly improved PFS (HR, 0.68; 95% CI, 0.56-0.84; **Figure 3A**), OS (HR, 0.66; 95% CI, 0.57-0.77; **Figure 3B**), and ORR (OR, 1.73; 95% CI, 1.15-2.59; **Figure 3C**) in the PD-L1-positive population. Conversely, no significant statistical disparities were detected in the PD-L1-negative population (**Figure 3**). However, it is crucial to acknowledge the limited number of PD-L1-negative patients, which necessitates cautious interpretation of these data.

**Figure 4:**
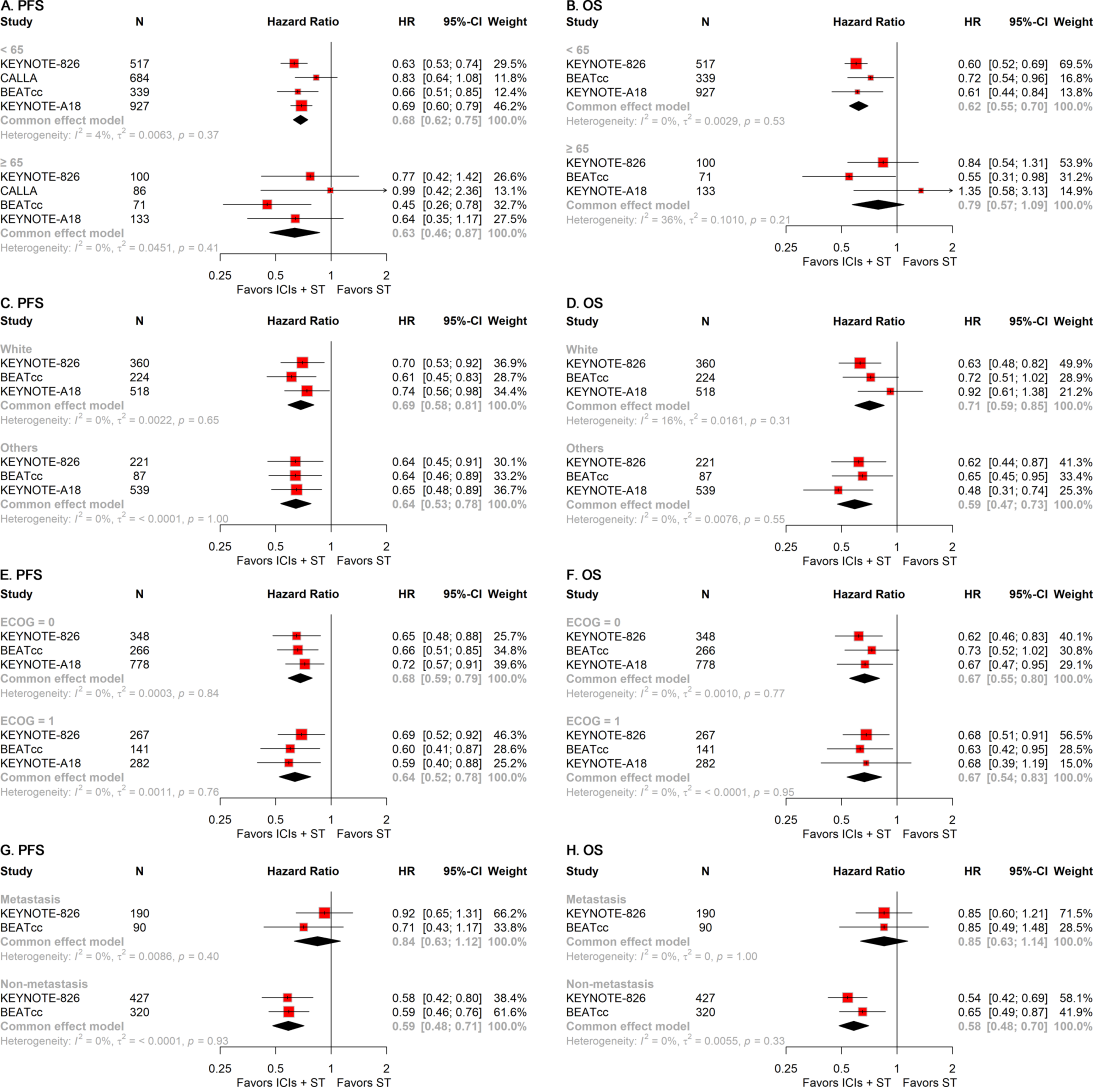
Forest plots of subgroup analysis stratified by clinical characteristics. Results for age on PFS **(A)** and OS **(B)**. Results for race on PFS **(C)** and OS **(D)**. Results for ECOG performance status on PFS **(E)** and OS **(F)**. Results for disease status on PFS **(G)** and OS **(H)**. PFS, progression-free survival; OS, overall survival; ECOG, Eastern Cooperative Oncology Group.

To examine the impact of clinical characteristic variations on the efficacy of ICIs plus ST in patients with r/a CC, we conducted multiple subgroup analyses based on patient attributes, including age, race, Eastern Cooperative Oncology Group (ECOG) Performance Status, and disease status. In the subgroup of patients under 65 years of age, the addition of ICIs to ST significantly improved PFS (HR, 0.68; 95% CI, 0.62-0.75; **Figure 4A**) and OS (HR, 0.62; 95% CI, 0.55-0.70; **Figure 4B**). In contrast, for patients aged 65 years and older, the addition of ICIs to ST significantly improved PFS (HR, 0.63; 95% CI, 0.46-0.87; **Figure 4A**), whereas no significant differences in OS were observed (**Figure 4B**). Subgroup analyses based on race and ECOG status indicated that the addition of ICIs to ST significantly improved both PFS and OS irrespective of patient race (White and others) (**Figures 4C, D**) and ECOG status (0 and 1) (**Figures 4E, F**). Among patients with metastatic disease, the addition of ICIs to ST did not significantly improve either PFS or OS (**Figures 4G, H**). However, in patients with non-metastatic disease, the addition of ICIs to ST significantly enhanced both PFS (HR, 0.59; 95% CI, 0.48-0.71; **Figure 4G**) and OS (HR, 0.58; 95% CI, 0.48-0.70; **Figure 4H**).

**Figure 5:**
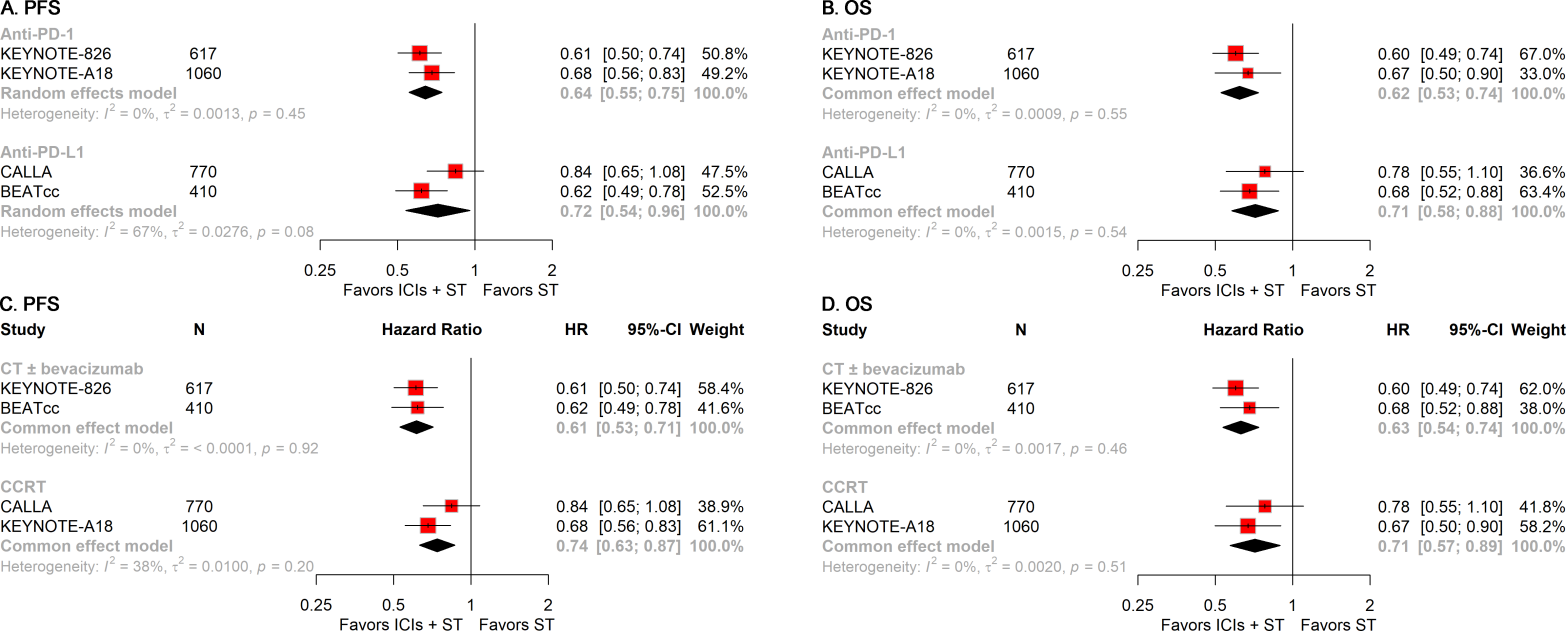
Forest plots of subgroup analysis stratified by treatment regimens. Results for the type of ICIs used on PFS **(A)** and OS **(B)**. Results for the regimens of ST employed on PFS **(C)** and OS **(D)**. PFS, progression-free survival; OS, overall survival; ICIs, immune checkpoint inhibitors; ST, standard therapy.

To gain further insights into the treatment modalities, we performed stratified analyses according to the type of ICIs and ST employed in the treatment regimens. The addition of either anti-PD-1 or anti-PD-L1 to ST was associated with significant improvements in PFS (anti-PD-1: HR, 0.64; 95% CI, 0.55-0.75; anti-PD-L1: HR, 0.72; 95% CI, 0.54-0.96; **Figure 5A**) and OS (anti-PD-1: HR, 0.62; 95% CI, 0.53-0.74; anti-PD-L1: HR, 0.71; 95% CI, 0.58-0.88; **Figure 5B**). Similarly, the inclusion of ICIs in ST was linked to superior PFS (CT ± bevacizumab: HR, 0.61; 95% CI, 0.53-0.71; CCRT: HR, 0.74; 95% CI, 0.63-0.87; **Figure 5C**) and OS (CT ± bevacizumab: HR, 0.63; 95% CI, 0.54-0.74; CCRT: HR, 0.71; 95% CI, 0.57-0.89; **Figure 5D**), regardless of the specific ST regimen used.

### Risk of bias and sensitivity analysis

Among the four trials, three were conducted as double-blind trials, whereas one was conducted as an open-label trial. Consequently, the open-label trial was assessed as having a high risk of performance bias, an unclear risk of detection bias, and a low risk of selection, attrition, and reporting biases. The remaining studies were all evaluated as having a low risk of bias across all assessed criteria. The risk of bias assessment is graphically summarized in **Supplementary Figure S1**. The funnel plot, along with Egger’s test (*P* = 0.2984), did not indicate significant publication bias (**Supplementary Figure S2**). Sensitivity analysis for PFS, OS, and ORR confirmed the robustness of the pooled results (**Supplementary Figure S3**).

## Discussion

Currently, the integration of ICIs into ST has emerged as a predominant area of research for patients diagnosed with r/a CC. However, published phase 3 trials have yielded conflicting results, leading to ongoing debate regarding the efficacy of ICIs combined with ST in treating r/a CC. The KEYNOTE-826 trial is a landmark study that evaluated pembrolizumab in conjunction with platinum-based CT and/or bevacizumab. The results demonstrated a significant improvement in both PFS and OS compared to CT alone. The CALLA trial explored the addition of durvalumab to CCRT; unfortunately, the study did not meet its primary endpoints compared to CCRT alone. The BEATcc trial investigated the incorporation of atezolizumab into a regimen of bevacizumab plus CT. The results showed a significant improvement in PFS and OS compared to CT and bevacizumab alone. In parallel, the ENGOT-cx11/GOG-3047/KEYNOTE-A18 trial assessed the efficacy of adding pembrolizumab to CCRT for r/a CC. The findings revealed a notable enhancement in both PFS and OS for patients receiving the combination therapy compared to those treated with CCRT alone. Therefore, we included these four phase 3 trials encompassing a total of 2,857 participants to evaluate the efficacy and safety of ICIs in combination with ST as a first-line treatment for patients with r/a CC. Our findings provide high-quality, evidence-based recommendations for the clinical management of patients with r/a CC and highlight crucial considerations for treating patients with different PD-L1 statuses.

While ICIs plus ST offer promising efficacy, it is important to acknowledge the increased toxicity associated with this treatment approach. Our meta-analysis revealed a slight increase in grade 3-5 AEs with ICIs plus ST compared to ST alone. These findings highlight the importance of carefully monitoring and managing AEs in patients undergoing ICIs plus ST. Clinicians should weigh the potential benefits of ICIs plus ST against the risk of increased toxicity when considering this treatment option for r/a CC patients.

To gain deeper insights into the efficacy of ICIs plus ST in the treatment of r/a CC, we conducted several specific subgroup analyses focusing on PD-L1 status, clinical characteristics, and treatment regimens. These subgroup analyses suggest that the combination of ICIs and ST holds particular therapeutic promise in the following patient populations: i) PD-L1-positive patients. The combination therapy of ICIs and ST demonstrated a significant improvement in patients with PD-L1-positive r/a CC, indicating that PD-L1 status is a critical biomarker for identifying patients who are likely to benefit the most from this treatment approach. ii) Patients aged less than 65 years. Our data revealed that the benefits of ICIs plus ST in terms of PFS and OS were more pronounced in patients under the age of 65. This suggests that younger patients may have a more favorable response to this combination therapy. iii) Non-metastatic patients. The combination of ICIs and ST appeared to be more effective in patients with non-metastatic disease compared to those with metastatic disease. This finding highlights the potential for ICIs to improve outcomes in patients with less advanced forms of the disease. These findings have significant therapeutic implications, as they can guide clinicians in personalizing treatment strategies for r/a CC patients. By focusing on these specific populations, clinicians can optimize the use of ICIs and ST, potentially leading to improved patient outcomes and minimizing the risk of unnecessary treatment-related AEs in those who may not benefit as much.

The potential of CCRT to enhance anticancer immune responses by promoting the release of cancer antigens is widely recognized (16). However, the optimal CCRT regimen to synergize with ICIs remains an open question, prompting ongoing research to identify treatment strategies that effectively mobilize and activate tumor-specific T cells while mitigating immune suppression. The results from the KEYNOTE-A18 study did not align with those observed in the CALLA study, highlighting the need for further investigation into the interplay between CCRT and ICIs in r/a CC. Three possibilities may explain the differences observed between CALLA and KEYNOTE-A18: i) Differences in drugs. Durvalumab is a PD-L1 inhibitor, while pembrolizumab is a PD-1 inhibitor. This raises the question of whether a PD-1 antibody targeting the T-cell surface has a more direct regulatory effect on the immune system compared to a PD-L1 antibody targeting the tumor cell surface, potentially leading to better treatment outcomes (17). ii) Patient population. The CALLA study enrolled a relatively high proportion of patients with early-stage disease (IB2-IIB). Patients with early-stage disease generally experience favorable outcomes with CCRT alone, which could narrow the survival gap between experimental and control groups, making it difficult to observe significant statistical differences in PFS between the two study arms (18). iii) Radiation dose regimen. The radiation dose regimen specified in the CALLA study might be more conservative compared to KEYNOTE-A18. Clinically, achieving a tumor-killing dose (radical dose) with radiotherapy is crucial for local control and patient prognosis (19). Unlike CT, immunotherapy may not achieve satisfactory tumor control as monotherapy (20). Therefore, adding immunotherapy to a regimen with an insufficient tumor-killing dose may not fully exploit the long-lasting benefits of immunotherapy. These considerations underscore the complexity of integrating CCRT with ICIs and highlight the necessity for further research to optimize treatment strategies for r/a CC.

There are some limitations in this meta-analysis. Firstly, a discrepancy exists in the techniques utilized to assess PD-L1 across the trials. Specifically, two trials employed the PD-L1 IHC 22C3 pharmDx assay, characterizing PD-L1 expression by a combined positive score (CPS) ≥ 1 (9, 10, 13). In contrast, the CALLA trial assessed PD-L1 expression according to the tumor area positivity (TAP) score using the VENTANA PD-L1 (SP263) assay, with TAP ≥ 1% serving as the criterion (11). This discrepancy in PD-L1 assessment poses a significant challenge in clinical studies exploring immunotherapy for r/a CC. To address this issue, there is a need for better harmonization of PD-L1 testing across clinical trials to ensure consistency and comparability of results. Secondly, the reporting of AEs was inconsistent across the included studies, and only those AEs reported in all trials were included in this meta-analysis. This limits our comprehensive understanding of potential AEs associated with the treatment regimens.

## Conclusion

This meta-analysis is the inaugural study to elucidate that the integration of ICIs into ST represents a potent and relatively low-risk therapeutic strategy for individuals with r/a CC, offering robust support for the management of this malignancy. The synergistic effect of ICIs and ST is particularly pronounced within certain subsets of patients, including those with high PD-L1 expression, those younger than 65 years, and those with a non-metastatic disease state. The assessment of PD-L1 expression serves as a valuable biomarker in identifying patients likely to experience enhanced therapeutic gains from the combined regimen of ICIs and ST. The implications of these findings for clinical decision-making are significant, highlighting the need for further research to optimize the integration of ICIs with ST in the treatment of r/a CC. As such, these data have the potential to inform future clinical guideline development, particularly with regard to the incorporation of ICIs into standard ST protocols.

## Data Availability

The original contributions presented in the study are included in the article/**Supplementary Material**. Further inquiries can be directed to the corresponding author.
