# Supplementary material for: Efficacy and safety of adding immune checkpoint inhibitors to first-line standard therapy for recurrent or advanced cervical cancer: a meta-analysis of phase 3 clinical trials
Source: Front Immunol. 2024 Dec 6;15:1507977. doi: 10.3389/fimmu.2024.1507977 (PMC11659232; doi:10.3389/fimmu.2024.1507977)
Supplement: Supplementary file 1 [file DataSheet1.docx]

**Supplementary Material**

**Efficacy and safety of adding immune checkpoint inhibitors to standard therapy for recurrent or advanced cervical cancer: A meta-analysis of phase 3 clinical trials**

**Fig. S1.** Results of the assessment of risk of bias.

**Fig. S2.** Funnel plot and Egger’s test for progression-free survival.

**Fig.S3.** Sensitivity analyses for included studies on overall survival, progression-free survival, and objective response rate examined by leave-one-out approach.

**
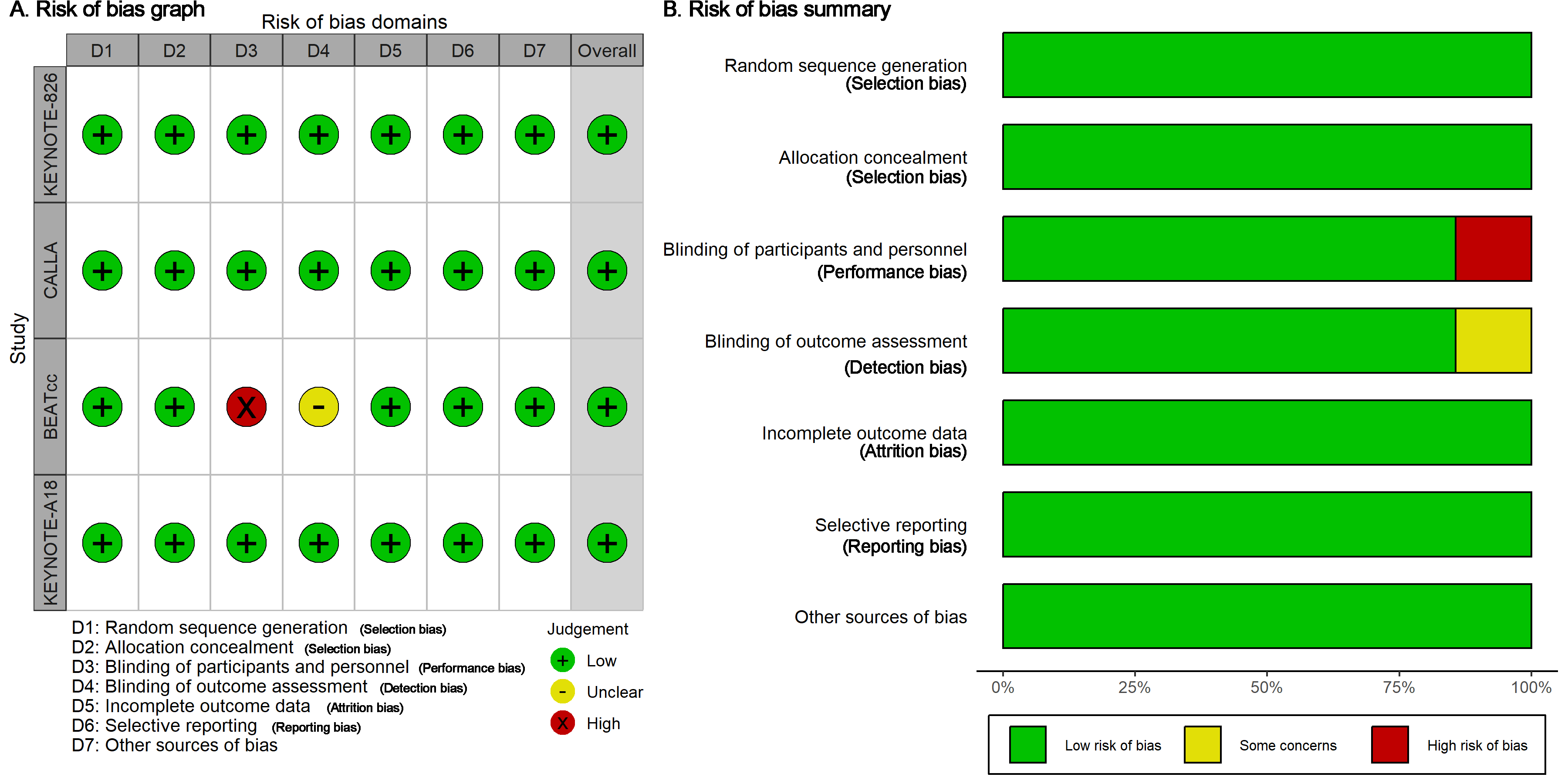
**

**Fig. S1.** Results of the assessment of risk of bias.

**
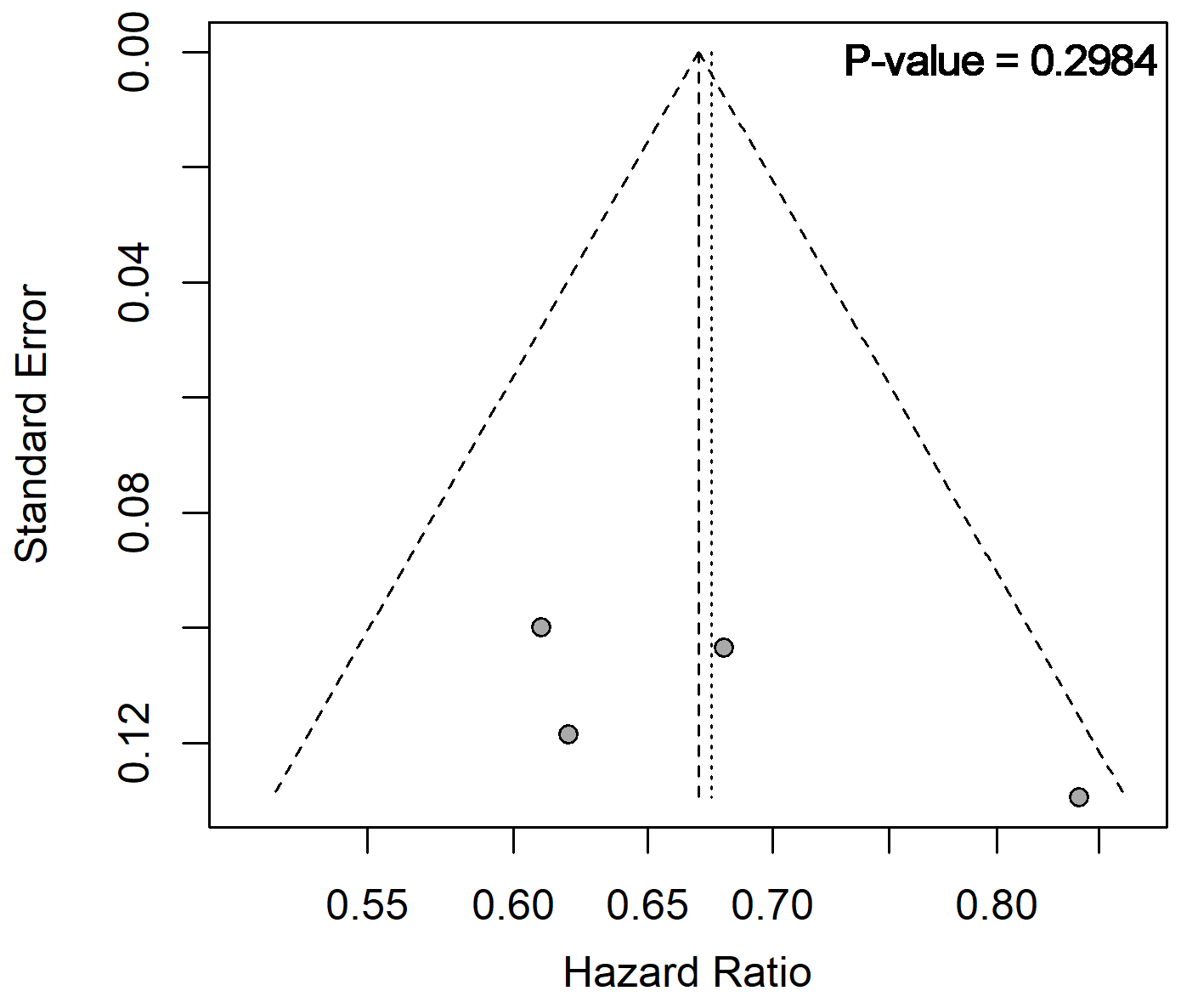
**

**Fig. S2.** Funnel plot and Egger’s test for progression-free survival.

**
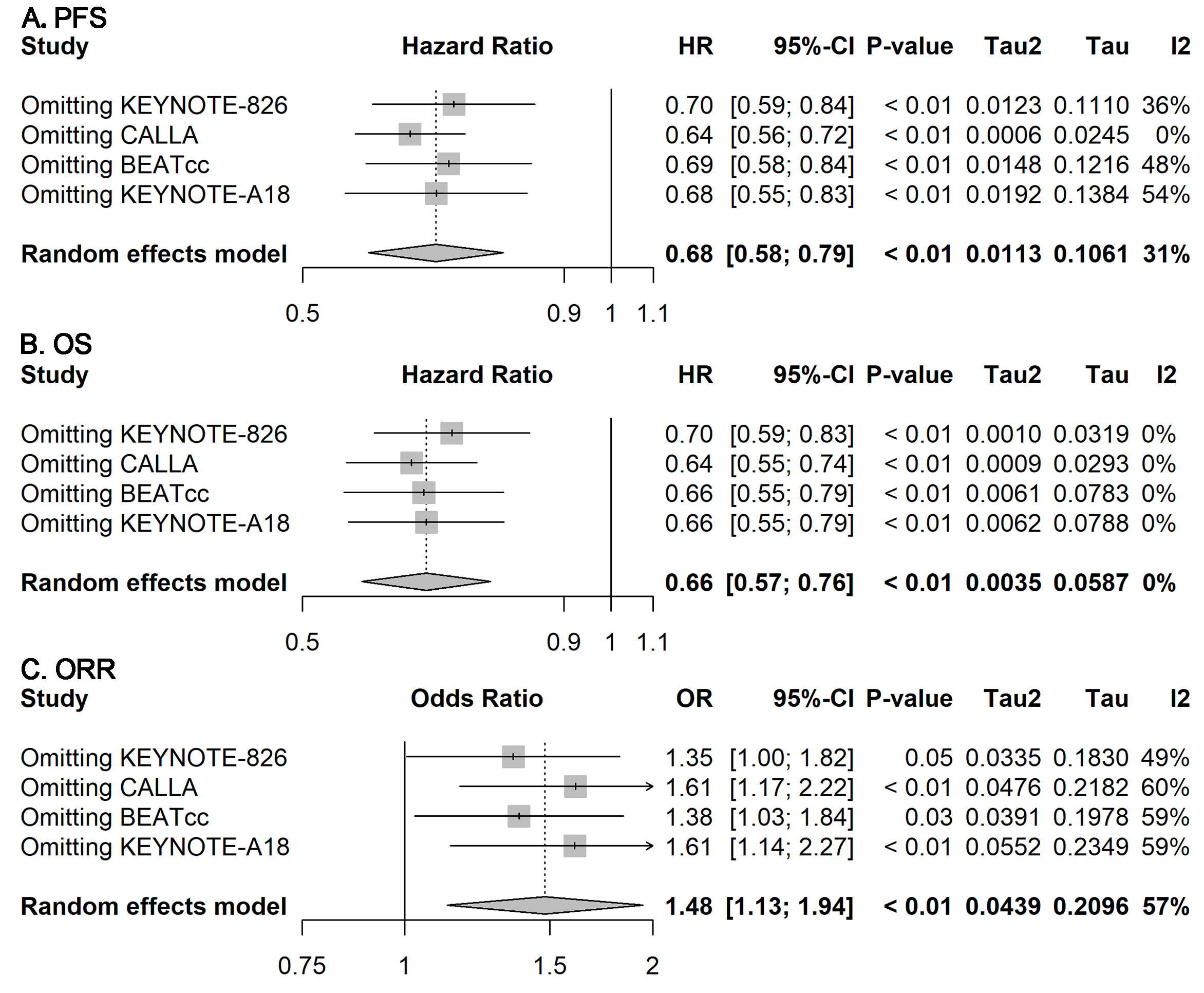
**

**Fig. S3** Sensitivity analyses for included studies on PFS, OS, and ORR examined by leave-one-out approach. OS, overall survival; PFS, progression-free survival; ORR, objective response rate.
